# Toxic Effects of Fumonisins, Deoxynivalenol and Zearalenone Alone and in Combination in Ducks Fed the Maximum EUTolerated Level

**DOI:** 10.3390/toxins13020152

**Published:** 2021-02-16

**Authors:** Céline Peillod, Marie Laborde, Angélique Travel, Amandine Mika, Jean Denis Bailly, Didier Cleva, Cyril Boissieu, Jean Le Guennec, Olivier Albaric, Sophie Labrut, Pascal Froment, Didier Tardieu, Philippe Guerre

**Affiliations:** 1 ITAVI, Centre INRA Val de Loire, 37380 Nouzilly, France; PEILLOD@maisadour.com (C.P.); Laborde@Itavi.Asso.Fr (M.L.); travel@itavi.asso.fr (A.T.); mika@itavi.asso.fr (A.M.); 2 Equipe Biosynthèse et toxicité des mycotoxines, ENVT, UMR Toxalim, Université de Toulouse, F-31076 Toulouse, France; jean-denis.bailly@envt.fr; 3 Chêne Vert Conseil, Z Bellevue II, 35220 Chateaubourg, France; d.cleva@cavac.fr (D.C.); c.boissieu@chenevertconseil.com (C.B.); 4 Finalab, 4 bis rue Th. Botrel, BP 351, 22603 Loudéac CEDEX, France; j.leguennec@finalab.fr; 5 ONIRIS, Site de la Chantrerie, BP 40706, 44307 Nantes CEDEX 3, France; oalbaric@gmail.com (O.A.); sophie.labrut@oniris-nantes.fr (S.L.); 6 Equipe GCR INRA–Physiologie de la Reproduction et des Comportements-UMR INRA-CNRS (UMR 6175)-Université François Rabelais de Tours, 37380 Nouzilly, France; pascal.froment@inrae.fr; 7 ENVT, Université de Toulouse, F-31076 Toulouse, France; didier.tardieu@envt.fr

**Keywords:** ducks, fumonisins, deoxynivalenol, zearalenone, interactions

## Abstract

Toxic effects among fumonisins B (FB), deoxynivalenol (DON) and zearalenone (ZEN) administered alone and combined were investigated in 84-day-old ducks during force-feeding. 75 male ducks, divided into five groups of 15 animals, received daily during the meal a capsule containing the desired among of toxin. Treated animals received dietary levels of toxins equivalent to 20 mg FB1+FB2/kg (FB), 5 mg DON/kg (DON), 0.5 mg ZEN/kg (ZEN) and 20, 5 and 0.5 mg/kg of FB, DON and ZEN (FBDONZEN), respectively. Control birds received capsules with no toxin. After 12 days, a decrease in body weight gain accompanied by an increase in the feed conversion ratio was observed in ducks exposed to FBDONZEN, whereas there was no effect on performances in ducks exposed to FB, DON and ZEN separately. No difference among groups was observed in relative organ weight, biochemistry, histopathology and several variables used to measure oxidative damage and testicular function. A sphinganine to sphingosine ratio of 0.32, 1.19 and 1.04, was measured in liver in controls and in ducks exposed to FB and FBDONZEN, respectively. Concentrations of FB1 in liver were 13.34 and 15.4 ng/g in ducks exposed to FB and FBDONZEN, respectively. Together ZEN and its metabolites were measured after enzymatic hydrolysis of the conjugated forms. Mean concentrations of α-zearalenol in liver were 0.82 and 0.54 ng/g in ducks exposed to ZEN and FBDONZEN, respectively. β-zearalenol was 2.3-fold less abundant than α-zearalenol, whereas ZEN was only found in trace amounts. In conclusion, this study suggests that decreased performance may occur in ducks exposed to a combination of FB, DON and ZEN, but does not reveal any other interaction between mycotoxins in any of the other variables measured.

## 1. Introduction

Mycotoxins are secondary fungal metabolites present in a wide range of raw materials used in food and feed. Because of their fungal origin, the occurrence of mycotoxins varies considerably depending on the raw material, the country of origin and the year of production. Although recent investigations revealed that food contamination by aflatoxins is a new emerging risk due to climate change, fusariotoxins (FUS) are the most abundant mycotoxins found in cereals in Europe [1,2]. Poultry are particularly exposed to FUS because of the high proportion of cereals in their diets [3,4]. Because of their toxicity, regulatory guidelines on FUS in cereals and poultry feed have been established [5,6]. However, although a major problem of feed contamination by mycotoxins is linked to the number of compounds present, most studies on FUS toxicity in avian species have been based on the effects of each toxin administered separately and do not take their possible combined effects into consideration. Unfortunately, multiple contamination is the most frequent scenario and contamination of mycotoxins at levels that individually should have no effects, may affect the animals when fed in combination due to synergistic or additive toxicity [1,7]. In addition, most guidelines for poultry species originate from studies on broilers and turkeys, which are the most frequently raised species, whereas studies on ducks revealed that this species is highly sensitive to FUS, particularly fumonisin B (FB) [8,9,10].

Recent studies in chickens and turkeys fed the maximum EU tolerated level of fumonisins B (FB), deoxynivalenol (DON) and zearalenone (ZEN), alone and in combination, showed no effect of interactions among these toxins on performances, biochemistry, histopathology, oxidative damage and testis toxicity [11,12]. But because chickens and turkeys are known for their relatively high tolerance to FUS in their diet, the lack of toxic interaction does not mean these interactions do not occur in other species [13,14,15,16,17,18,19,20,21,22,23,24,25,26,27,28]. In addition, studies on chickens and turkeys suggest that multiple contamination of feed by FUS could alter the persistence of the toxins in tissues and that these changes may differ with the toxin and the species [29,30]. Because ducks are more sensitive to the toxic effects of FUS than broilers and turkeys, a study of the toxic effects and persistence of FB, DON and ZEN, alone and in combination in ducks, could reveal interactions that have not been observed in chickens and turkeys [9,10,31,32,33,34]. The interest of a study on ducks is particularly justified at force-feeding due to a high corn intake during this period, which can lead to high exposure of the animals to mycotoxins [3,9].

The objective of this work was thus to investigate the toxic effects of FB, DON and ZEN, administered alone and in combination to male ducks on a large number of markers of performances and toxicity. Biomarkers of exposure to and of the persistence of FB and ZEN and its metabolites were also investigated. The toxins were administered to each bird to insure all the animals received the same concentration of each substance.

## 2. Results

### 2.1. Performances and Organ Weight

No mortality and no clinical signs that could be attributed to mycotoxicosis were observed in the ducks over the course of the 12-day study. Table 1 compares the performances of ducks not exposed to FUS and ducks orally exposed to mycotoxins equivalent to 20 mg FB1+FB2/kg (FB), 5 mg DON/kg (DON), 0.5 mg ZEN/kg (ZEN) and 20, 5 and 0.5 mg/kg of FB, DON and ZEN (FBDONZEN), respectively. At 96 days of age (D96), body weight (BW) was significantly lower in ducks exposed to FBDONZEN for 12 days than in the other groups. This effect went with a lower daily weight gain (DWG) whereas no difference among groups occurred on feed consumption (FC). The feed conversion ratio (FCR), obtained by dividing the body weight gain by the feed consumption over the entire period, was higher in ducks exposed to FBDONZEN than in all the other groups (Table 1).

The effects of FUS on the weight of liver, heart, spleen, pancreas and Fabricius bursa and on the melt rate of the liver are also listed in Table 1. A significant difference among groups was observed in kidney weight. Comparison of means revealed that kidney weights were lower in ducks exposed to FBDONZEN than in ducks fed the control diet, whereas kidney weight did not differ in any of the other groups. Although a numerical decrease in the liver weight was also observed in ducks exposed to FBDONZEN, the difference among groups was not significant (ANOVA, *p* = 0.0891). By contrast, no differences were found among groups in the relative organ weights of kidney and liver (data not shown). The effects of FUS on the weights and the length of some sections of the gastro digestive tract were also measured. Although significant differences in duodenum weight, in the jejunum and in ileum length were observed among groups (ANOVA), none of the groups exposed to FUS differed from the group of control ducks not exposed to FUS.

### 2.2. Biochemistry and Histopathology

Table 2 shows the biochemistry and hematology variables measured in control ducks and in ducks orally exposed to FUS. No differences in the concentration of proteins, cholesterol, uric acid and the activity of lactate dehydrogenase (LDH), alkaline phosphatase (ALP) and alanine aminotransferase (ALT) were observed among groups. Similarly, no differences in heterocytes, lymphocytes and monocytes were observed among groups.

The results of histopathological analyses of the liver, kidney, spleen and Fabricius bursa are reported in Table 2. Examination of the liver revealed vacuolization of hepatocytes but with no differences among groups. Polymorphic periportal infiltrates consisting mainly of lymphocytes were observed at minimum to low intensity in the livers of all the groups. Some livers also showed slight hyperplasia of the bile ducts unrelated to the feed ingested. Low intensity interstitial infiltration by heterophilic granulocytes and lymphocytes was observed in most kidneys. Acidophilic droplets associated with red blood cells or desquamated cells were observed in a limited number of animals, unrelated to the feed distributed. Lymphoid hyperplasia of mild to moderate intensity was observed in the spleens, whereas a slight to moderate multifocal lymphoid depletion of the bursa of Fabricius was observed. These lesions were not of toxicological significance and were not differently distributed among groups.

Lesions in the cecum and caecum tonsils are also reported in Table 2. Mild to moderate typhlitis was observed in all the animals. These lesions are characterized by the presence of an inflammatory infiltrate of lymphocytes and plasma cells accompanied by hyperplasia of the lymphoid tissue normally present in the cecum wall. No dilatation of the cecum glands was observed. One-way ANOVA revealed significant differences in the inflammation of the cecum and in the infiltration of cecum tonsils among the groups, but Kruskal–Wallis comparison of means failed to reveal a significant difference among groups.

### 2.3. Oxidative Markers and Testis Function

Table 3 lists the different variables used to investigate oxidative damage and the activities of the enzymes engaged in the defense against such toxic effects in plasma and liver. Together, the levels of malondialdehyde (MDA), total glutathione (TGs) and oxidized glutathione (GSSG) and the activities of superoxide dismutase (SOD), catalase (CAT), glutathione peroxidase (GPx) and glutathione reductase (GRed) were measured. The concentration of GSSG and both SOD and CAT activities were too low to enable their measurement in plasma. No differences in any of the variables measured in plasma and in liver were found among groups.

The different markers used to measure testis integrity are also reported in Table 3. The weight of the testis and the activity of cleaved caspase 3 were similar in all the groups. Similarly, no differences in cAMP and testosterone contents in testis were observed among groups.

### 2.4. Sphingolipids and Mycotoxins in Liver

**Figure 1:**
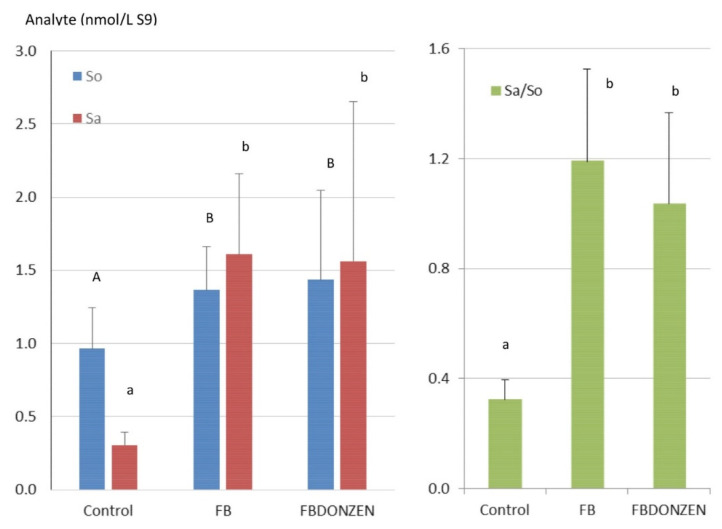
Sphinganine (Sa), sphingosine (So) and Sa:So ratio (Sa/So) in liver of control ducks not exposed to mycotoxins and in duck administered fumonisins B (FB) alone at a dose equal to 20 mg FB1+FB2/kg diet (FB) and FB combined with deoxynivalenol and zearalenone (FBDONZEN) at respective concentrations of 20, 5 and 0.5 mg/kg. ANOVA revealed a significant difference (*p* < 0.05) among groups. A Kruskal–Wallis comparison of means test was performed, different letters identify statistically different groups (*p* < 0.05). Results are presented as mean ± SD (*n* = 14).

The levels of sphinganine (Sa), sphingosine (So) and the Sa:So ratio measured in the liver of ducks are shown in Figure 1. Mean concentrations of So in the ducks not exposed to FUS and in ducks orally exposed to FB and FBDONZEN in combination were 0.96, 1.36 and 1.44 nmol/L, respectively. Concentrations of Sa measured in the same samples were 0.3, 1.61 and 1.56 nmol/L, respectively. Sa:So ratios were 0.32, 1.19 and 1.04, in control ducks and in ducks exposed to FB and to FBDONZEN, respectively. One-way ANOVA revealed significant differences among groups in both So and Sa concentrations as well as in the Sa:So ratio in the liver. Kruskal–Wallis comparison of means revealed that the levels of So and Sa and the Sa:So ratios were higher in ducks exposed to FB and FBDONZEN in combination than in control ducks. The means of Sa, So and Sa:So observed in ducks exposed to FB alone did not differ from those observed in ducks exposed to FB in combination with DON and ZEN.

**Figure 2:**
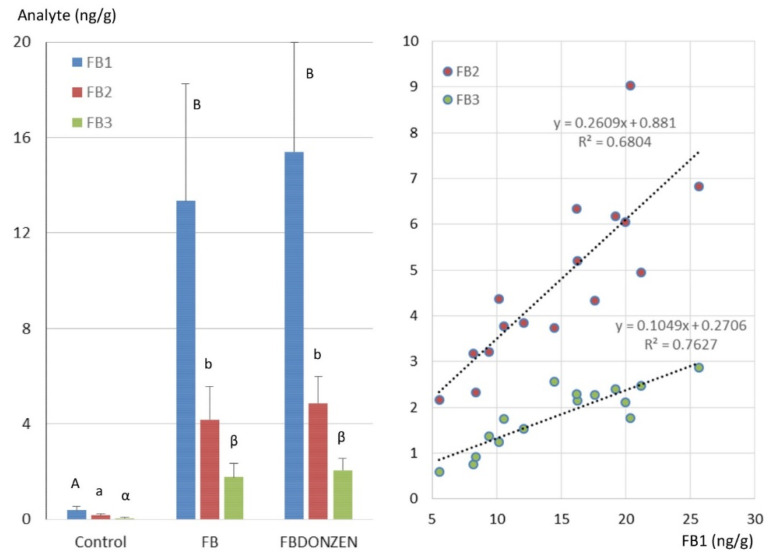
Fumonisins concentrations in liver of control ducks not exposed to mycotoxins and in ducks administered fumonisins B alone at a dose equal to 20 mg FB1+FB2/kg diet (FB) and FB combined with deoxynivalenol and zearalenone (FBDONZEN) at respective concentrations of 20, 5 and 0.5 mg/kg. Results are presented as mean ± SD (*n* = 8). ANOVA revealed significant differences (*p* < 0.05) among groups. A Kruskal–Wallis comparison of means test was performed, different letters identify statistically different groups (*p* < 0.05). The right side of the figure shows the linear regressions between the concentrations of FB1 and the concentrations of FB2 and FB3 measured in the livers of ducks exposed to FB.

Figure 2 shows the levels of FB dosed in the liver eight hours after the last distribution of feed. Eight liver samples were analyzed in each group. The level of FB1 in the liver of animals not exposed to FUS was less than 1 ng/g, in all the analyzed samples except one, in which very low level of FB1 (1.08 ng/g) was quantified. The concentrations of FB2 and FB3 in all the liver samples of animals orally not exposed to FB were below 0.5 ng/g. The mean levels of FB1, FB2 and FB3 in the liver of ducks exposed to FB were 13.58, 4.66 and 1.64 ng/g, respectively. The mean levels of FB1, FB2 and FB3 in the livers of ducks exposed to FBDONZEN were 15.48, 4.78 and 1.99 ng/g, respectively. Kruskal–Wallis comparison of means revealed no difference between ducks exposed to FB alone and ducks exposed to FBDONZEN. A linear regression analysis of the relationship between the concentrations of FB1 and FB2 or FB3 measured in the livers of ducks exposed to fumonisins showed that the concentrations were significantly correlated (Pearson, *p* < 0.05) with R^2^ values of 0.6804 and 0.7627, respectively. The steering coefficients of the regression lines established between the concentrations of FB1 and FB2 or FB3 were 0.261 and 0.105, respectively.

**Figure 3:**
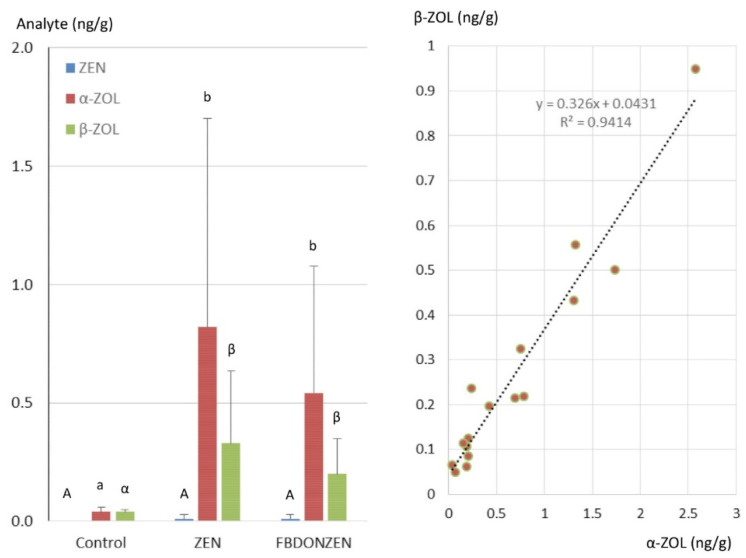
Zearalenone (ZEN), α-zearalenol (α-ZOL) and β-zearalenol (β-ZOL) concentrations in liver of control ducks not exposed to mycotoxins and in ducks administered ZEN alone at a dose equal to 0.5 mg/kg diet (ZEN) and ZEN combined with fumonisins and deoxynivalenol (FBDONZEN) at respective concentrations of 20, 5 and 0.5 mg/kg. Results are presented as mean ± SD (*n* = 8). ANOVA revealed significant differences (*p* < 0.05) among groups. A Kruskal–Wallis comparison of means test was performed, different letters identify statistically different groups (*p* < 0.05). The right side of the figure shows a linear regression between the concentrations of α-ZOL and β-ZOL measured in the liver of ducks exposed to ZEN.

ZEN and its metabolites were measured in the liver of ducks not exposed to FUS and in ducks orally exposed to ZEN at 0.5 mg/kg feed alone and in combination (Figure 3). Eight liver samples were analyzed in each group. Whatever the sample analyzed, ZEN was not detected or only found in trace amounts, while α-ZAL and β-ZAL were not detected. In addition, α-ZOL and β-ZOL were below 0.1 ng/g in the liver of ducks not exposed to FUS. By contrast the mean levels of α-ZOL in the liver of ducks exposed to ZEN and FBDONZEN were 0.88 and 0.54 ng/g, respectively, four samples being above the LOQ in each group. β-ZOL was less abundant than α-ZOL and was at 0.33 and 0.2 ng/g, in the liver of ducks exposed to ZEN and FBDONZEN, respectively. Kruskal–Wallis comparison of means revealed that the levels of α-ZOL and β-ZOL observed in ducks exposed to ZEN alone did not differ from those observed in ducks exposed to ZEN combined with FB and DON. A linear regression analysis of the relationship between the concentrations of α-ZOL and β-ZOL measured in the livers of ducks exposed to zearalenone, showed that the concentrations were significantly correlated (Pearson, *p* < 0.05) with an R^2^ value of 0.9414. The steering coefficient of the regression line established between the concentrations of α-ZOL and β-ZOL was 0.326.

## 3. Discussion

### 3.1. Performances, Organs Weight, Biochemistry and Histopathology

No mortality and no clinical signs that could be attributed to mycotoxicosis were observed in the ducks in this study. No change on performances or on organ weight was observed in ducks exposed to FB, DON and ZEN alone at a concentration equivalent to 20, 5 and 0.5 mg/kg feed, respectively. This result was surprising because previous studies conducted with FB at a concentration of 20 mg FB1/kg feed and 20 mg FB1+FB2/kg feed in ducks revealed a slight increase in mortality and a marked reduction in the weight of the liver [9,10]. Vacuolization of hepatocytes was observed in all the livers, which is common in ducks, whereas, surprisingly, no difference in the size of the vacuoles was observed among groups. This result disagrees with the results of a previous study which revealed that steatosis was due to the formation of macro vesicles of fat in control ducks while micro vesicles were observed in ducks fed with 20 mg FB1/kg [9]. Differences between the studies are discussed below by accounting for the systemic exposure of the animals that can be assessed by measuring the Sa:So ratio and the concentration of FB1 in liver.

Exposure to FB combined with DON and ZEN reduced performance and kidney weight compared to controls. This result is the first to suggest that a synergic interaction between FUS could occur in ducks and contrary to with results obtained in chickens and turkeys that did not demonstrate any interaction between FB, DON and ZEN [11,12]. Differences between the studies could be due to the greater sensitivity of ducks to mycotoxins, especially FB. Interestingly, the decrease in kidneys weight observed in ducks exposed to FUS in a mix in this study was accompanied by a decrease in BW, whereas no significant difference in the relative kidney weight was observed among the ducks. This observation suggests that the decrease in kidney weight is probably more related to the decrease in BW rather than to a toxic effect of FUS on the kidney. This hypothesis was reinforced by the results of the biochemical and histopathological analyses of organs that revealed no difference between ducks exposed to FUS and control ducks. The slight inflammation observed in most of the ducks was not related to the administration of FUS and had no toxicological significance. The lack of interaction among FUS on biochemistry and histopathology is in agreement with results obtained in broilers and turkeys [11,12].

### 3.2. Oxidative Markers and Testis Function

The measure of oxidative damage and the determination of the activities of the enzymes involved in the defense against such toxic effects in the plasma and in the liver did not show any effect of FUS alone or in combination. This observation is in agreement with previous results in broilers and turkeys [11,12]. Our results confirm that hepatic steatosis occurs in ducks during gavage and is not accompanied by oxidative damage, suggesting that the mechanisms involved in steatosis in ducks are significantly different from those observed in other species. [35,36,37].

In the present study, no significant differences in the markers of testis integrity were found among groups. Caspase 3, which is used as a marker of apoptosis and cAMP and testosterone contents, which are used as markers of testis functions, were not affected by FUS either alone or in combination. This result agrees with the results of studies conducted in male broilers and male turkeys [11,12]. It shows that reproductive function of male ducks is relatively resistant to FUS toxicity, which is in agreement with the rare data in the literature on the effects of FUS on the male reproductive performance in avian species [4,38].

### 3.3. Sphingolipids and Mycotoxins

Sa and the Sa:So ratio in liver were considerably higher in ducks exposed to FB than in control ducks not exposed to FB. This result agrees with data previously obtained in ducks fed FB [9,10] and confirms that the Sa:So ratio is a valuable biomarker of FB exposure in ducks [39,40,41]. Concomitant exposure to DON and ZEN plus FB did not alter the Sa:So ratio, suggesting that DON and ZEN did not interfere in the effects of FB on Sa:So. A recent study conducted in chickens reported a slight decrease in the Sa:So ratio in animals fed DON and ZEN combined with FB compared to chickens fed FB alone, whereas a study conducted in turkeys suggested the contrary [11,12]. Differences between the studies could be due to the exact doses of FB to which the animals were exposed. In the present study, FB was individually administered to each animal in capsules given during a meal, to be sure all the ducks received exactly the same FB dose. By contrast, contaminated diets were used in studies conducted in chickens and turkeys, which led to a variation of around 25% in FB contents. Slight differences in the amount of feed consumed among groups could contribute to the apparent interactions observed between FUS on the Sa:So ratio in the studies conducted in turkeys and chickens [11,12].

FB1, FB2 and FB3 were above the LOQ in the liver of ducks exposed to 20 mg FB1+FB2/kg feed. This result confirms that FB1 can be found in the liver of different poultry species exposed to FB in diets at the maximum tolerated level defined by the EU [10,29,42,43,44]. The feed:liver ratio of FB observed in ducks agrees with previous works conducted in this animal species and was below than the feed:liver ratio of FB observed in chickens and turkeys [10,29,42,43]. The concomitant exposure to DON and ZEN combined with FB did not alter the concentration of FB found in liver compared to ducks exposed to FB alone. This result supports previous observations made in chickens and turkeys [29].

Concerning ZEN and its metabolites, α-ZOL was the most abundant analyte found in duck liver in this study. The ratio of α-ZOL to β-ZOL was approximately 2.5, while ZEN was only measured in trace amounts and α-ZAL and β-ZAL were not found. These results are consistent with previous data obtained in poultry species [45]. The levels of α-ZOL and β-ZOL in duck liver are very close to those observed in chickens when ZEN was administered at 0.6 mg/kg of feed for 14 days but lower than those observed in turkeys when ZEN was administered at 0.5 or 0.6 mg/kg of feed for 14 days and lower than those observed in chickens when ZEN was administered at 0.5 mg/kg of feed for 35 days [30,44]. A strong correlation was observed between α-ZOL and β-ZOL in ducks, which agrees with previous results observed in turkeys and chickens [30,44]. The concomitant exposure to FB and DON combined with ZEN did not alter the concentration of ZEN and its metabolites found in liver compared to ducks exposed to ZEN alone. This result disagrees with previous observations made in chickens and turkeys in which the concomitant exposure to FB and DON combined with ZEN increased the levels of ZEN and its metabolites in the liver [30]. Differences between the studies could be due to the animal species and the amounts of ZEN and its metabolites present in the liver.

In conclusion, this study suggests a decrease in performances may occur in ducks exposed to a combination of FB, DON and ZEN, but revealed no interactions in biochemistry, histopathology, markers of oxidative damage and markers of testis function. Sa, the Sa:So ratio and FB1 in the liver are thus useful biomarkers of FB exposure in ducks, while α-ZOL in the liver is an useful biomarkers of ZEN exposure in ducks. The concomitant exposure of animals to DON and ZEN did not alter the Sa:So ratio or the persistence of FB1 and α-ZOL in the liver.

## 4. Materials and Methods

### 4.1. Chemicals and Reagents

All chemicals and reagents were acquired from Scharlau (Scharlau Chimie S.A., Barcelona, Spain), Carlo Erba (Carlo Erba, Val de Rueil 27, France) and Sigma Chemical Co (Sigma, Saint Quentin Fallavier, France). The Bio-Rad kit were purchased from Bio-Rad Laboratories (Biorad, München, Germany). The DC assay kit were purchased from Uptima Interchim (Interchim, Montluçon, France). Caspase 3/7 Glow assays and the cAMP-Glo Assay were purchased from Promega (Promega, Charbonnieres les bains, France). Enzymatic hydrolysis of ZEN conjugates and its metabolites was performed by the H-2 β-glucuronidase from *Helix pomatia* acquired from Sigma (Cat# G0875). FB1, FB2, FB3, ZEN, α-ZOL, β-ZOL, ZAN, α-ZAL, β-ZAL, DON, DON-3-glucoside, deepoxy-DON, 15-acetyl-DON, 3-acetyl-DON, fusarenone x, nivalenol, diacetoxyscirpenol, 15 monoacetoxyscirpenol, T2 toxin, HT2 toxin, T2 tetraol, verrucarol, roridin A, verrucarin A, moniliformin, tenuazonic acid, ergocornine, ergocristine, ergocryptine, ergometrine, ergosine, ergotamine, aflatoxin B1, aflatoxin B2, aflatoxin G1, aflatoxin G2, ochratoxin A, ochratoxin B, alpha-ochratoxin, cyclopiazonic acid, citrinin, patulin and sterigmatocystin were purchased as standards from Biopure (Romer Labs, Getzersdorf, Austria) and Sigma. Solutions of [^13^C_34_]-FB1, [^13^C_34_]-FB2, [^13^C_34_]-FB3 and [^13^C_18_]-ZEN at certified concentrations were acquired from Biopure (Romer Labs, Tulln, Austria). Sphinganine, sphingosine and C20 sphinganine were acquired from BioValley (BioValley S. A., Marne la Vallée, France). FUMONIPREP and EASI-EXTRACT^®^ ZEARALENONE columns were acquired from R-Biopharm Rhône Ltd. (R-Biopharm AG, Glasgow, Scotland). Pure water, acetonitrile, methanol, formic acid, ammonium acetate and acetic acid used for analysis of mycotoxins were of LC-MS analytical grade whereas other reactants were of HPLC analytical grade.

### 4.2. Production of Mycotoxins

Fumonisins were obtained from culture of strain L12 of *Fusarium verticillioides* on crushed corn (aw 0.99) at 25 °C. Deoxynivalenol and zearalenone were obtained from culture of strain I159 of *F. graminearum* on wheat at 23 °C and from strain I171 on rice at 21 °C, respectively. The cultivation period lasted four weeks then the cultured material was dried for 3 h at 90 °C, ground and sieved in a 0.6 mm mesh sieve. The level of mycotoxins in the grounded culture material was measured by HPLC-MSMS. Each ground material was diluted with a corn flour free of mycotoxins to reach respective concentrations of 14.3, 1.14 and 0.273 mg/g of FB1+FB2, DON and ZEN. The diluted ground culture medium was placed in capsules of different sizes to reach mycotoxin exposure equivalent to the ingestion of a feed containing 20, 5 and 0.5 mg/kg of FB1+FB2, DON and ZEN, respectively (Table S1). Concomitant exposure to FB, DON and ZEN was accomplished by administering capsules containing the different toxins. The concentration of 20 mg FB1+FB2/kg corresponded to the sum of 15.25 and 4.75 mg/kg of FB1 and FB2, respectively.

### 4.3. Mycotoxins in Culture Material and Corn

The levels of mycotoxins in the grounded culture material and in the corn used to fed ducks were analyzed by HPLC-MSMS according the AFNOR V03-110 guideline as previously described [11]. Separation of the analytes was done on a C_18_ phase column (VWR Pessac, France) using an HPLC Hewlett Packard 1100 type (Hewlett Packard, Eybens, France), while detection of the analytes was done with a quadrupole tandem mass spectrometer API 4000 (Applied Biosystems, Foster City, CA, USA). The mobile phase was composed of ammonium acetate 1nM and 0.0001% acetic acid/methanol and 1% acetonitrile. A linear gradient was applied at a flow rate of 1 mL/min for 40 min. Detection of mycotoxins was done in positive and negative mode at source temperature 500 °C and 4500 V ion spray voltage. Two or three transitions were used for each mycotoxin. One kg of corn or 50 g of culture material that contained the mycotoxins was ground to a fine powder (0.5 mm). An aliquot of 5 g was collected and 20 mL of acetonitrile/water (vol:vol) were added. After two hours of extraction on a stir table, the sample was centrifuged and 3 mL of the upper phase were collected and evaporated to dryness. The dry residue was solubilized in 0.01% acetic acid/methanol (2:1) and filtered on a syringe prior to injection in the LC-MS/MS system. Depending on the mycotoxin assayed, the limit of quantification ranged from 1 to 10 µg/kg.

### 4.4. Animal Exposure

The study was conducted in agreement to the guidelines of the Declaration of Helsinki and the French National Guidelines for the care and use of animals for research purposes. Protocol was approved by the institutional review board UEPFG of INRAE (protocol code PP–5–12 approved in date of February 29, 2012). Seventy-five male mule ducks (*Cairina moschata* × *Anas platyrhynchos*) from the commercial lines MMG × PKL (Couvoir Ducournau, Bonnegarde, France) were reared in individual cages at the Palmipole station (Domaine d’Artiguères, Benquet, France). From 0 to 84 days of age, the ducks were fed with mycotoxin-free diets to best meet their nutritional needs. On the 84th day of age, the ducks were weighed and distributed to form five weight-homogeneous groups each containing 15 birds to be exposed to FUS. The force-feeding program lasted 12 days and followed the usual practice in the species [46]. The corn was administered as a mixture of 26% whole corn, 36% ground corn and 38% water as follow: 250 g of corn on the first meal, followed by a progressive increase of 25 g per meal to reach a volume of 500 g of corn per meal on day 6, which was maintained until the end of the study (Table S1). Capsules containing FB, DON and ZEN were administered in the middle of the meal to the ducks exposed to FUS while mycotoxin-free capsules were administered to the control animals not exposed to mycotoxins. Each animal received two meals per day. At the end of the study, the animals were left to fast for eight hours, then a blood sample was taken from the post-occipital sinus of the spinal vein on lithium heparin vacutainer tubes before ducks were stunned by electro narcosis and killed by exsanguination.

### 4.5. Sample Collection

All the animals were autopsied for macroscopic investigation of outward appearance of the eyes, digestive system (including beak and accessory glands), kidneys and the musculoskeletal, respiratory, cardiovascular and reproductive systems to identify anomalies. Organs (liver, kidneys, heart, spleen, pancreas and testes) were collected and the intestine was emptied prior to isolation of the different fractions (gizzard, duodenum, jejunum, ileum and caeca, including caeca tonsils). All organs and digestive fractions were weighed and the length of the duodenum, jejunum and ileum was measured. Tissue fractions of organs and intestine were collected and stored in neutral buffered formalin for microscopic investigation while the remaining liver and testes were stored at −80 °C for other analysis.

### 4.6. Biochemistry, Hematology, Histopathology and Tissue Fractions

The concentrations of proteins, cholesterol and uric acid and the activities of lactate dehydrogenase (LDH, EC 1.1.1.27), alkaline phosphatase (ALP, EC 3.1.3.1) and alanine aminotransferase (ALT, EC 2.6.1.2) were measured in plasma with a clinical chemistry analyzer KONELAB 20 (Fisher Scientific SAS, Illkirch, France) according to the manufacturer’s instructions. Concentrations in analytes were expressed in g/L or mmol/L and enzymes activities were expressed in UI/L. Malassez cells was used for manually counting of the number of white blood cell and results were expressed in %.

Fixed tissues were cut into fractions of about 1 cm and embedded in paraffin blocks. Paraffin blocks were cut into slices of 4 μm that were stained with hematoxylin, eosin and saffron (HES). Semi-quantitative analysis of lesions was measured for each tissue of each animal using the following score: absence = 0: slight = 1, moderate = 2, marked = 3.

S9 homogenates of liver were obtained by milling 5 g of liver in 15 mL of PBS as previously described [11]. Five hundred microliters of S9 were deproteinized with 1.25 M metaphosphoric acid (vol:vol). The testis extracts were obtained by three consecutive freeze/thaw cycles in PBS. S9 fractions, deproteinized S9 and testis extracts were stored at −80 °C until analysis.

Melt rate of the liver was measured as previously described [47]. Briefly, 60 g of liver were placed in a tin and cooked in an autoclave at 85 °C for 60 min. After refrigeration, the tins were heated to 75–80 °C, 20 min and the melting rate was measured as the percentage of melted fat in proportion to the initial weight of the liver.

### 4.7. Markers of Oxidative Damage and Analysis of Testis Extracts

Markers of oxidative damage in plasma and S9 liver fractions and analysis of testis extracts were investigated as previously described [11]. Briefly, the concentration of protein in the S9 homogenate and in the testis, the extract was determined with the Bio-Rad protein kit and the DC assay kit, respectively. Malondialdehyde (MDA) was measured by formation of a pink complex with thiobarbituric acid that was extracted by butanol and fluorometrically dosed (excitation at 515 nm, emission at 548 nm). The activity of superoxide dismutase (SOD, EC 1.15.1.1) was measured in the presence of nitroblue tetrazolium by the inhibition of the formation of blue formazan by 1 mU xanthine-xanthine oxidase (EC 1.17.3.2). The reaction was monitored spectrophotometrically at 540 nm. The activity was obtained by linear regression using a standard curve performed with bovine erythrocyte SOD. The activity of catalase (CAT, EC 1.11.1.6) was measured with H_2_O_2_ as substrate by the formation of formaldehyde, which was spectrophotometrically dosed at 540 nm. The activity was obtained by linear regression using a standard curve performed with formaldehyde. The activity of glutathione reductase (GRed, EC 1.6.4.2) was measured by reduction of oxidized glutathione (GSSG) into glutathione (GSH) in the presence of NADPH. The activity was obtained by following the decrease in NADPH absorbance at 340 nm. The activity of glutathione peroxidase (GPx, EC 1.11.1.9) was measured in a coupled reaction with GRed. In the presence of GSH, GPx reduces cumene hydroperoxide to produce GSSG. Then, GSSG is reduced into GSH by GRed in the presence of NADPH. The activity of the enzyme was obtained by following the decrease in absorbance at 340 nm. The deproteinized S9 fraction is used to measure total glutathione (TG) using an optimized enzymatic recycling method for the determination of GSH content. GSH reacts with Ellman’s reagent to form 5-thio-2-nitrobenzoic acid (TNB) and GS-TNB. Both GSSG and GS-TNB are reduced by GRed to produce GSH, permitting the formation of more TNB. The amount of TNB produced was spectrophotometrically measured at 405 nm. The GSSG amountis obtained by a first derivatization of GSH with 2-vinylpyridine before measurement of TGs, which block the reaction of GSH.

Caspase 3/7 Glow assays were performed using the Caspase-Glo^®^ 3/7 Assay System kit of Promega (Promega, Charbonnieres les Bains, France) according to the manufacturer’s instructions. The activity of caspase-3/7 is obtained by using a luminogenic substrate containing the sequence asp-glu-val-asp that liberates luciferin after cleavage by caspases. Luciferin is used by luciferase to generate luminescence that was expressed in relative light units normalized to 100,000 seminiferous tubules cells. The concentration of cAMP was measured using the cAMP-Glo^®^ Assay kit of Promega as recommended by the manufacturer. The concentration of testosterone was determined by radioimmunoassay with a sensitivity of 15 pg/tube and 5.3% intra-assay coefficients of variation.

### 4.8. Sphingosine and Sphinganine in Liver

Free sphingoid bases sphingosine (So) and sphinganine (Sa) were measured by HPLC as previously described [39]. Briefly, C20 sphinganine was added as internal standard (IS) to S9 homogenates to a final concentration of 2 nmol/mL. Alkaline methanolic-chloroform was added to the homogenate to hydrolyze the lipids and liberate the sphingoïd bases. After two washing with alkaline water, the chloroform phase was evaporated to dryness. The dried residue was suspended in ethanol and placed in an insert. Derivatization of sphingoid bases was done by an automate using orthophtaldialdehyde. Ten µL were injected into the HPLC system composed of an ICS M2200 solvent delivery module (ICS, Toulouse, France) connected to a RF10 AXL fluorescence detector (Shimadzu, Kyoto, Japan). The analytes were separated on a Prontosil C18 column (250 × 4.6 mm) coupled to a C18 precolumn (Bischoff, Leonberg, Germany). The liquid phase was composed of methanol-water (90:10) delivered at a flow rate of 1.25 mL/min. Detection was performed at excitation wavelength of 335 nm and emission wavelength of 440 nm. Concentrations of the sphingoïd bases were obtained by linear regression from standard solutions and corrected by the rate of recovery measured on C20 sphinganine was used as IS.

### 4.9. Mycotoxins in Liver

The concentration of mycotoxins in the liver was analyzed by UHPLC-MSMS after sample clean-up on immunoaffinity column and isotopic dilution, as previously described [29,30]. The UHPLC MSMS system was a 1260 model, the detector was a 6410 triple quad, both from Agilent (Agilent, Santa Clara, CA, USA). Mycotoxins were separated on a Poroshell 120 column (3.0 × 50 mm, 2.7 µ). Detection was done in positive electrospray ionization mode in the following conditions: temperature at 300 °C, gas delivered at 10 L/min, nebulization at 25 psi and capillary voltage at 4000 V. Two transitions were used as qualifiers for FB1, FB2, FB3, ZEN, α-ZOL, β-ZOL, ZAN, α-ZAL and β-ZAL, whereas one qualifier was used for the internal standards. MassHunter quantitative analysis software (Agilent) was used to analyze the chromatograms. A relative standard deviation of 20% was considered acceptable for measuring accuracy. The signal suppression and enhancement (SSE) was calculated by dividing the slope of calibration curve fitted to matrix by the slope of calibration curve based on net solvent and expressed in percent. Values of SSE of 80–120% were considered acceptable. The maximum variation of the ratio of the qualifiers measured in the samples had to be less than 20% of the ratio measured in the standard solutions. The maximum variation in the retention time of the analytes in the samples had to be less than 5% of the retention time measured in the standard solutions.

The method used to measure FB in liver was previously described [29]. Briefly, one g of liver was homogenized in 4 mL of distilled water acetonitrile/methanol (2:1:1) in presence of 5 mg of NaCl and 12.5 ng of [^13^C_34_]-FB1 and [^13^C_34_]-FB2 as IS. Samples were extracted 2h on a stir table, then centrifuged. Aqueous phase was collected and 8 mL of hexane were added for removing the lipids. Part of the aqueous phase was passed through a FUMONIPREP column. Extracts were evaporated to dryness and stored at −20 °C. The method was linear (Fisher test, *p* < 0.01 and r2 ≥ 0.99) for a concentration range of 0.25 to 25 ng/g for FB1, FB2 and FB3, the lowest concentrations validated corresponded to the LOQ [29].

The method used to measure the total forms of ZEN and its metabolites in liver was previously described [30]. Briefly, five g of liver were homogenized in 5 mL of acetate buffer in presence of 1000 U of H-2 β-glucuronidase and placed overnight in a shaking bath at 37 °C. The mixture was placed in a conical tube containing of 12.5 ng of [^13^C_18_]-ZEN and thirty ml of acetonitrile/hexane (2:1) were added. Samples were placed 15min on a stir table, then centrifuged. The upper phase was removed and the lower phase was centrifuged again to collect the supernatant that was evaporated to dryness. The dry residue was suspended in 2.5 mL methanol; then, 22.5 mL of PBS was added and the solution was passed through the Easi-Extract Zearalenone Column. Extracts were evaporated to dryness and stored at −20 °C. The method was linear (Fisher test, *p* < 0.01 and r2 ≥ 0.99) for a concentration range of 0.25 to 4 ng/g for α-ZOL and β-ZOL and 1 to 4 ng/g for ZEN; the lowest concentrations validated corresponded to the LOQ [30].

### 4.10. Statistical Analysis

The statistical analyses were performed using XLSTAT- LIFE SCIENCES - Biomed from Addinsoft (Addinsoft, Bordeaux, France). Data are reported as means ± SD or SEM. One-way ANOVA was performed to compare groups when the test used to check the homogeneity of variance passed (Hartley’s test) while a *t*-test was used for variables that revealed heterogeneity of variance. When significant differences among groups (*p* < 0.05) were observed, a comparison of means was conducted with Duncan’s multiple range test or Kruskal–Wallis test. Different letters were used to identify statistically different groups (*p* < 0.05) in a same row. Correlations between analytes found in the liver were performed using Pearson’s correlation coefficient test.

## Figures and Tables

**Table 1:** Effects of fusariotoxins (FUS) on performance and organ weights in ducks ^1^.

Variable ^3^	Control ^2^	FB ^2^	DON ^2^	ZEN ^2^	FBDONZEN ^2^
D84 BW (g)	4696 ± 89	4691 ± 84	4690 ± 86	4699 ± 85	4693 ± 87
D96 BW (g) *	6656 ± 293 ^a^	6620 ± 402 ^a^	6545 ± 362 ^a^	6648 ± 289 ^a^	6289 ± 278 ^b^
DWG (g) *	163 ± 20 ^a^	161 ± 28 ^a^	155 ± 24 ^a^	162 ± 20 ^a^	133 ± 21 ^b^
FC	9115 ± 297	9210 ± 244	9236 ± 257	9129 ± 401	9165 ± 350
FCR *	4.71 ± 0.56 ^a^	4.90 ± 0.77 ^a^	5.07 ± 0.67 ^a^	4.74 ± 0.56 ^a^	5.90 ± 1.08 ^b^
Liver (g)	697 ± 125	646 ± 136	640 ± 140	672 ± 126	567 ± 125
Melt rate (%)	0.41 ± 0.07	0.35 ± 0.15	0.37 ± 0.10	0.35 ± 0.16	0.30 ± 0.15
Kidney (g) *	28.4 ± 2.4 ^a^	27.9 ± 2.8 ^ab^	28.5 ± 3.5 ^a^	27.4 ± 1.7 ^ab^	25.6 ± 2.9 ^b^
Heart (g)	34.4 ± 4.4	33.3 ± 4.1	36.1 ± 4.4	33.9 ± 4	33.5 ± 4
Spleen (g)	2.73 ± 0.64	2.51 ± 0.56	2.79 ± 0.48	2.62 ± 0.55	2.56 ± 0.51
Pancreas (g)	9.75 ± 1.13	9.42 ± 1.70	9.87 ± 1.17	9.60 ± 1.55	9.93 ± 1.74
F. bursa (g)	1.77 ± 0.55	1.83 ± 0.51	1.63 ± 0.34	1.74 ± 0.53	1.75 ± 0.61
Gizzard (g)	72.9 ± 8.6	73.1 ± 8.2	73.1 ± 6.9	72.8 ± 8.6	72 ± 7.8
Intestine (g)	119 ± 17	120 ± 23	118 ± 26	112 ± 20	105 ± 20
Intestine (cm)	277 ± 5	289 ± 18	282 ± 22	274 ± 20	269 ± 21
Duodenum (g) *	25.4 ± 4.2 ^ab^	22.9 ± 3.9 ^ab^	25.9 ± 5 ^a^	24 ± 4 ^ab^	21.3 ± 4 ^b^
Duodenum (cm)	47.1 ± 4.3	45.4 ± 3.8	47.8 ± 4	45.6 ± 2.4	46.1 ± 6
Jejunum (g)	51.7 ± 9.4	54.3 ± 12	52 ± 12.5	48.8 ± 9.5	45.4 ± 10.4
Jejunum (cm) *	116 ± 8 ^ab^	123 ± 9 ^a^	120 ± 9 ^ab^	117 ± 11 ^ab^	113 ± 9 ^b^
Ileum (g)	42.1 ± 7.7	42.9 ± 8.7	39.9 ± 10	39.4 ± 7.3	38.7 ± 8.2
Ileum (cm) *	114 ± 8	121 ± 10	115 ± 12	113 ± 10	110 ± 9
Caecum (g)	3.27 ± 0.69	3.63 ± 1.25	3.46 ± 0.67	3.25 ± 0.51	3.41 ± 0.67

^1^ Values are mean ± SD, *n* = 14. One-way ANOVA was performed to compare groups. When a significant difference was observed (*, *p* < 0.05), a Kruskal–Wallis comparison of means test was used. Different letters in the same row identify statistically different groups (*p* < 0.05). ^2^ FUS were administered in capsules during a meal to obtain an exposure equivalent to the ingestion of a feed containing 20 mg FB1+FB2/kg (FB), 5 mg DON/kg (DON), 0.5 mg ZEN/kg (ZEN) and 20, 5 and 0.5 mg/kg of FB, DON and ZEN (FBDONZEN), respectively. ^3^ D84, D96 = days of age 84 and 96, respectively; BW = body weight; DWG = daily weight gain; FC = feed consumption; FCR = feed conversion ratio; F. bursa = Fabricius bursa; DWG and FCR were calculated from D84 to D96.

**Table 2:** Effects of FUS on biochemistry, hematology and histopathology in ducks ^1^.

Variables ^3^	Control ^2^	FB ^2^	DON ^2^	ZEN ^2^	FBDONZEN ^2^
Proteins ^3^	41.4 ± 10.8	42.7 ± 6.7	43.4 ± 10.9	42.7 ± 12.2	48.6 ± 14.4
Cholesterol ^3^	3.41 ± 1.11	3.44 ± 0.83	3.57 ± 0.87	3.5 ± 0.74	3.79 ± 0.99
Uric acid ^3^	82.8 ± 28.8	83.8 ± 27.9	84 ± 19.4	88.6 ± 25.2	80.9 ± 20.8
LDH ^3^	7474 ± 3085	6060 ± 1986	6786 ± 2844	7310 ± 1905	7025 ± 3329
ALP ^3^	4.91 ± 1.45	5.64 ± 1.55	5.64 ± 1.34	6.00 ± 2.63	5.71 ± 1.86
ALT ^3^	168 ± 91	145 ± 69	133 ± 83	120 ± 42	123 ± 43
Heterocytes ^4^	59.9 ± 14.6	44.1 ± 7.8	47.1 ± 13.6	47.9 ± 11.8	49.6 ± 10.7
Lymphocytes ^4^	49.6 ± 11.6	55.4 ± 7.8	51.9 ± 13.4	51.6 ± 11.7	49.9 ± 10.8
Monocytes ^4^	0.29 ± 0.61	0.57 ± 0.65	0.86 ± 1.10	0.50 ± 0.76	0.57 ± 0.65
Liver ^5^	0.4 ± 0.5	0.4 ± 0.5	0.4 ± 0.5	0.4 ± 0.5	0.7 ± 0.5
Kidney ^5^	0	0	0.1 ± 0.3	0.3 ± 0.6	0.1 ± 0.4
Spleen ^5^	1.5 ± 0.5	1.5 ± 0.5	1.5 ± 0.6	1.4 ± 0.5	1.4 ± 0.5
F. bursa ^5^	0.6 ± 0.9	0.4 ± 0.7	0.5 ± 0.9	1 ± 1	0.9 ± 0.9
G. hypertrophy ^6^	0.3 ± 0.5	0	0.3 ± 0.5	0.4 ± 0.5	0.5 ± 0.5
Inflammation ^6,^*	1.2 ± 0.8	1.7 ± 0.5	2 ± 0.1	1.9 ± 0.4	1.9 ± 0.3
L. hyperplasia ^6^	1.2 ± 0.4	1.6 ± 0.9	2 ± 0.6	1.9 ± 0.8	1.8 ± 0.4
C.T. infiltration ^6,^*	0.8 ± 0.4	0.5 ± 0.5	0.9 ± 0.6	1 ± 0.4	0.9 ± 0.3

^1^ Values are mean ± SD (*n* = 14). One-way ANOVA was performed to compare groups. When a significant difference was observed (*, *p* < 0.05), a Kruskal–Wallis comparison of means test was used, but no difference among groups was observed (*p* > 0.05). ^2^ FUS were administered in capsules during a meal to obtain an exposure equivalent to the ingestion of a feed containing 20 mg FB1+FB2/kg (FB), 5 mg DON/kg (DON), 0.5 mg ZEN/kg (ZEN) and 20, 5 and 0.5 mg/kg of FB, DON and ZEN (FBDONZEN), respectively. ^3^ Results are expressed in the following units: Proteins, g/L; Cholesterol, mmol/L, Uric acid, mmol/L; Lactate dehydrogenase (LDH), UI/L; Alkaline phosphatase (ALP), UI/L; Alanine aminotransferase (ALT), UI/L. ^4^ Heterocytes, lymphocytes and monocytes measured on Malassez cells and expressed in %. ^5^ Main lesions scored for the different organs were liver: polymorphic periportal infiltrates; kidney: medullary interstitial infiltration; spleen: lymphoid hyperplasia; Fabricius bursa: multifocal lymphoid depletion. ^6^ Lesions observed on caecum that corresponded to glandular hypertrophy (G. hypertrophy); Inflammation; lymphoid hyperplasia (L. hyperplasia); cecum tonsils infiltration by heterophilic granulocytes (C.T. infiltration).

**Table 3:** Effects of FUS on oxidative stress and testis integrity in ducks ^1^.

	Control ^2^	FB ^2^	DON ^2^	ZEN ^2^	FBDONZEN ^2^
Plasma ^3^					
MDA	983 ± 248	1105 ± 177	995 ± 209	1070 ± 181	1111 ± 293
TG	13.9 ± 1.8	11.7 ± 3.9	10.9 ± 3.9	10.6 ± 2.5	8.7 ± 2.7
GPx	493 ± 48	509 ± 33	515 ± 44	496 ± 44	509 ± 36
GRed	10.6 ± 4.4	16.7 ± 3.2	16 ± 3.5	15.8 ± 4	16.4 ± 4.1
Liver ^3^					
MDA	2.5 ± 1.7	3.6 ± 1.9	3.1 ± 1.7	3.6 ± 1.9	3.4 ± 1.9
TGs	22.2 ± 2.9	22.9 ± 4.4	21.6 ± 4.3	19.9 ± 2.6	20.2 ± 3.5
GSSG	1.7 ± 0.4	1.2 ± 0.4	1.1 ± 0.3	1.2 ± 0.5	1.5 ± 0.4
SOD	2.09 ± 1.04	1.95 ± 0.58	1.75 ± 0.7	1.69 ± 0.36	1.92 ± 0.59
CAT	23.4 ± 11.4	26.2 ± 13.7	24.3 ± 14.5	26.7 ± 11.1	27.7 ± 17.2
GPx	31.3 ± 6.1	30.4 ± 7.6	26.2 ± 6.3	31.4 ± 7.5	31 ± 5
GRed	12.7 ± 2.2	16 ± 2.1	14.2 ± 3.2	14.8 ± 4.3	15.7 ± 4.4
Testis ^4^					
Weight	2.08 ± 0.27	2.2 ± 0.33	1.87 ± 0.19	2.18 ± 0.26	2.07 ± 0.32
Caspase 3	0.9 ± 0.14	0.79 ± 0.13	1.15 ± 0.33	1.04 ± 0.21	0.76 ± 0.14
cAMP	164 ± 31	204 ± 21	195 ± 21	174 ± 29	167 ± 18
Testosterone	0.22 ± 0.05	0.29 ± 0.07	0.25 ± 0.08	0.31 ± 0.06	0.23 ± 0.06

^1^ One-way ANOVA was performed to compare groups. No significant difference between groups was observed (*p* > 0.05). ^2^ FUS were administered in capsules during a meal to obtain an exposure equivalent to the ingestion of a feed containing 20 mg FB1+FB2/kg (FB), 5 mg DON/kg (DON), 0.5 mg ZEN/kg (ZEN) and 20, 5 and 0.5 mg/kg of FB, DON and ZEN (FBDONZEN), respectively. ^3^ Results are expressed as mean ± SD per L of plasma or per mg of S9 liver proteins in the following units: Malondialdehyde (MDA) in nmol; Total glutathione (TG) in µmol GSSG; Oxidized glutathione (GSSG) in µmol; Superoxide dismutase (SOD) in U/min; Catalase (CAT) in µg of formaldehyde (CH_2_O)/min; Glutathione peroxidase (GPx) in µmol nicotinamide adenine dinucleotide phosphate (NADPH)/min; Glutathione reductase (GRed) in µmol NADPH/min. ^4^ Results are expressed as mean ± SEM (*n* = 14) in the following units: weight in g; Caspase 3 in relative luminescence units/mg proteins; cAMP in mM/mg proteins; Testosterone in ng/mg proteins.

## Data Availability

Data is contained within the article or supplementary materials.

## Supplementary Materials

The following are available online at https://www.mdpi.com/2072-6651/13/2/152/s1, Table S1: Amount of corn and amount of toxins administered to ducks.

## Abbreviations

EU	European Union
FUS	Fusariotoxins
FB	Fumonisins B
FB1	Fumonisin B1
FB2	Fumonisin B2
ZEN	Zearalenone
ZAN	Zearalanone
α-ZOL	Alpha-zearalenol
α-ZAL	Alpha-zearalanol
β-ZAL	Beta-zearalanol
β-ZOL	Beta-zearalenol
DON	Deoxynivalenol
SSE	Signal suppression and enhancement
Sa	Sphinganine
So	Sphingosine
BW	Body weight
DWG	Daily weight gain
FC	Feed consumption
FCR	Feed conversion ratio
LDH	Lactate dehydrogenase
ALP	Alkaline phosphatase
ALT	Alanine aminotransferase
MDA	Malondialdehyde
TGs	Total glutathione
GSSG	Oxidized glutathione
SOD	Oxidized glutathione
CAT	Catalase
GPx	Glutathione peroxidase
GRed	Glutathione reductase
IS	Internal standard
LOQ	Limit of quantitation
