# Supplementary material for: Toxic Effects of Fumonisins, Deoxynivalenol and Zearalenone Alone and in Combination in Ducks Fed the Maximum EUTolerated Level
Source: Toxins (Basel). 2021 Feb 16;13(2):152. doi: 10.3390/toxins13020152 (PMC7920068; doi:10.3390/toxins13020152)
Supplement: Supplementary file 1 [file toxins-13-00152-s001.pdf]

toxins

MDPI

Supplementary Materials: Toxic Effects of Fumonisins, Deoxynivalenol and Zearalenone Alone and in Combination in Ducks Fed the Maximum EU Tolerated Level

Céline Peillod, Marie Laborde, Angélique Travel, Amandine Mika, Jean Denis Bailly, Didier Cleva, Cyril Boissieu, Jean Le Guennec, Olivier Albaric, Sophie Labrut, Pascal Froment, Didier Tardieu and Philippe Guerre

Table S1. Amount of corn and amount of toxins administered to ducks [1].

			FB		DON		ZEN	
Day Number	N° of Meals	Corn (g)	FB1+FB2 [2]	Ground Material [2, 3]	DON [2]	Ground Material [3, 4]	ZEN [2]	Ground Material [3]
1	1	250	5	0.35	1.25	0.55	0.13	0.46
	2	275	5.5	0.38	1.38	0.6	0.14	0.5
2	3	300	6	0.42	1.5	0.66	0.15	0.55
	4	325	6.5	0.45	1.63	0.71	0.16	0.6
3	5	350	7	0.49	1.75	0.77	0.18	0.64
	6	375	7.5	0.52	1.88	0.82	0.18	0.69
4	7	375	7.5	0.52	1.85	0.82	0.19	0.69
	8	400	8	0.56	2	0.88	0.2	0.73
5	9	425	8.5	0.63	2.13	0.93	0.21	0.78
	10	450	9	0.66	2.25	0.99	0.22	0.82
6	11	475	9.5	0.68	2.38	1.04	0.24	0.87
	12	500	10	0.7	2.5	1.1	0.25	0.92
7	13	500	10	0.7	2.5	1.1	0.25	0.92
	14	500	10	0.7	2.5	1.1	0.25	0.92
8	15	500	10	0.7	2.5	1.1	0.25	0.92
	16	500	10	0.7	2.5	1.1	0.25	0.92
9	17	500	10	0.7	2.5	1.1	0.25	0.92
	18	500	10	0.7	2.5	1.1	0.25	0.92
10	19	500	10	0.7	2.5	1.1	0.25	0.92
	0	500	10	0.7	2.5	1.1	0.25	0.92
11	21	500	10	0.7	2.5	1.1	0.25	0.92
	22	500	10	0.7	2.5	1.1	0.25	0.92

[1] Capsules containing FB, DON, and ZEN were administered in the middle of the meal to the ducks exposed to FUS while mycotoxin-free capsules were administered to the control animals not exposed to mycotoxins. Ducks exposed to FBDON-ZEN received all three types of capsules containing FB, DON and ZEN; [2] in mg of toxin administered/meal/duck; [3] in g of ground material/capsule/duck. The capacity of the capsules used was 0.7, 1.1 and 0.916 g of ground material for FB, DON and ZEN, respectively. The capsules administered in meals 1 to 11 were completed with a corn flour free of mycotoxins to reach the targeted weight; [4] two capsules were administered/meal/duck.
